# Effect of co-administration of Bee honey and some chemotherapeutic drugs on dissemination of hepatocellular carcinoma in rats

**DOI:** 10.1016/j.toxrep.2019.08.007

**Published:** 2019-08-16

**Authors:** Naima Zayed Mohamed, Hanan Farouk Aly, Hatem Abdel moneim El-Mezayen, Hadeer E. El-Salamony

**Affiliations:** a Therapeutic Chemistry Department, Pharmaceutical and Drug Industries Research Division, National Research Centre (NRC), Giza, Egypt; b Biochemistry Department, Faculty of Science, Helwan University, Cairo, Egypt

**Keywords:** Hepatocellular carcinoma, Diethyl nitrosamine, Carbon tetrachloride, Bee honey, Chemotherapy, Oxidative stress, Inflammatory markers

## Abstract

Alternative and complimentary usage of the natural compound has raised hopes of finding curative options for liver hepatocarcinogenesis. In the present study, the curative effect of bee honey against diethylnitrosamine (DEN) (50 mg/kg) and carbon tetrachloride (CCl_4_) (2 mg/Kg)–induced hepatocellular carcinoma (HCC) in male rats in the presence or absence of some chemotherapeutic drugs, Cisplatin (Cis), Cyclophosphamide (CY) and 5- Fluorouracil (5-FU) were investigated. The obtained results demonstrated that treatment with DEN/CCl4 caused oxidative stress as assigned by the increase in malondialdehyde (MDA) and fall in glutathione (GSH) content. Meantime detraction in the antioxidants, including superoxide dismutase (SOD), catalase (CAT), glutathione-s-transferase (GST) and glutathione peroxidase (GPx) was observed. Also, the results showed induction of inflammation as reflected by an increase in the levels of both α- fetoprotein and α- fucosidase in the liver. This was accompanied by changes in the hepatic function biomarkers which characterized by the increased levels of transaminases (AST, ALT), alkaline phosphatase (ALP) and γ-Glutamyl transferase (γ-GT) and decrease in total protein content in the serum. In conclusion, the combination of the selected drugs and bee honey may be an effective chemo- preventive and therapeutic strategy for treating DEN and CCl4-induced HCC.

## Highlights

• Therapeutic effect of bee honey and three of the chemotherapeutic drugs were studied.
• Honey improved the effect of drugs and/or minimize their side effects.
• AFP, AFU and GGT are promising markers for HCC and treatment evaluation.

## Graphical abstract

**Figure:**
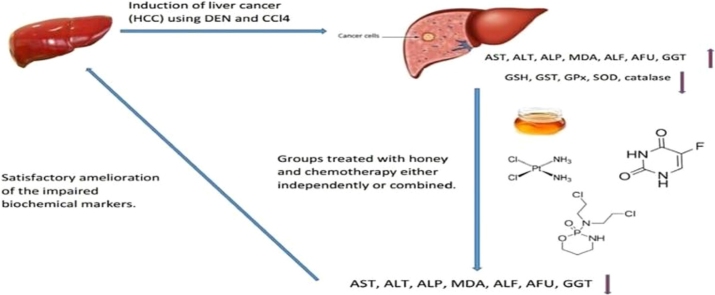

## 1 Introduction

The most common types of liver cancer in the world are the hepatocellular carcinoma (HCC). Also, it is considered as the second leading cause of cancer related deaths [1]. In Egypt, hepatocellular carcinoma is the second most logistical malignancy in males and fifth in females [2]. Chronic liver disease, especially cirrhosis is considered the vigorous adapting factor for the development of HCC [1]. Carcinogenic compounds such as aflatoxin and N-nitrosamines contaminated food [3] was also considered as a risk factor for HCC.

It is well known that in early stages of liver cancer, the main therapeutic option is partial hepatectomy. Although this surgery is proved to be effective and curative but post-surgery, liver cancer recurrence rates remain high, and hence further improvements in survival will require more effective therapeutic agents that might improve the results of resection [4].

The utmost acute hepatocarcinogensis in animals is N-nitrosamine compounds, especially diethyl nitrosamine (DEN) [5]. Diethyl nitrosamine is commonly used for HCC initiation; while CCl4 is introduced to enhance the intensity of carcinogenesis [6]. Oxidative stress is the output of production of reactive oxygen species and hepatocellular damage could be involved in the pathogenesis of DEN-induced hepatocellular carcinoma [5,7]. HCC development arises from the formation of alkyl DNA- DEN adducts and DEN induction of several nuclear aberrations in the rat liver [8].

Brown et al. [9] demonstrated that the inorganic molecule cisplatin, used in chemotherapy for various types of cancers. Cancer cell death occurred due to binding of cisplatin to DNA. Cisplatin also causes the lessening in the antioxidant situation and raise reactive oxygen species which lead to excess cytotoxicity [10]. Thence, cisplatin can cause adverse effects in a diversity of normal tissues, so its clinical use is bounded [11].

The alkylating agent cyclophosphamide (CP) is widely used in cancer chemotherapy [12]. Acrolein and phosphor amide are the two chemically reactive metabolites that produced in the liver arises from working CP. They slow the growth of cancer cells by interfering with the actions of deoxyribonucleic acid (DNA) within the cancerous cells [13]. Regrettably, normal cells also are affected, which gives rise to numerous side effects. Consequently, the application of CP for chemotherapy treatment is limited [14]. One of the wide applications to manage hepatocellular carcinoma (HCC) belongs to fluoropyrimidine family is 5-Fluorouracil (5-FU). Blocking of thymidylate synthase, the enzyme that catalyzes the de novo synthesis of the DNA precursor thymidylate is one of the most 5-FU mechanisms due to inhibition cell proliferation by forming fluorodeoxyuridine monophosphate. Also, the formation of defective F-RNA, which ultimately interferes with synthesis of protein to form defective, fluorinated DNA, was resulting in the breaking of the single-strand and fragmentation of DNA [[15], [16], [17]]. When 5-FU is taken by the cells it becomes toxic because it metabolized to fluoronucleotides which inserted into nucleic acids or bind to thymidylate synthase. Due to the rapid catabolism in the liver, blood, and other organs, the bioavailability of 5-FU is greatly limited. Inhibition of thymidylate synthetase by 5-FU and its metabolite 5-fluoro-2-deoxyuridine leads to blocking DNA synthesis [18]. Bhuvarahamurthy and Govindasamy [19] informed that the turmoil in collagen and mucopolysaccharide metabolism due to possible proteolytic enzyme unrest may lead to carcinoma tissue. HCC inducers like B or C viral infections, dietary exposure to aflatoxin B1 and chronic ethanol abuse or other genotoxic compounds such as tobacco smoke or nitrosamines from the diet are the main events to enter hepatocytes to hepatocarcinogensis [20,21]. Referable to the multiple etiologies and risk factors which define different pathways in hepatocarcinogensis, HCCs are heterogeneous [22]. There is no individual or combination chemotherapy regimen has been found to be especially effective in hepatocarcinogensis although great numbers of controlled and uncontrolled studies have been performed [22]. Systemic chemotherapy for hepatocellular carcinoma has been quite ineffective, despite the extensive attempts by many research workers. Until now, there is no regimen or drug that can be visibly determined as the standard for treating HCC.

Bee honey is an inbred output known for its assorted pharmacological and biological activities ranging from antioxidant, anti-inflammatory, and antihypertensive, hypoglycemic to antibacterial effects [23].

Recently, tyrosine kinase inhibitors had been approved as clinical strategy to treat cancer [24]. Honey and honey products were found to be effective as suppressors of tyrosine kinase activity and induction of cell cycle arrest in G1 or G2/M phase [25], and selective inhibition of cancerous cell viability [26,27]. This study focuses on the role of bee honey in modulating the outgrowth and advancement of hepatocellular carcinoma.

## 2 Material and methods

### 2.1 Materials

#### 2.1.1 Animals

Wistar male albino rats weighing (150 ± 30 g), were supplied from Animal House, National Research Centre (Dokki, Giza, Egypt), they were kept for one week to accommodate under constant environmental and nutritional conditions with free access to food and water. The protocol of experiment was approved by the Ethical Committee of Medical Division, National Research Centre, Egypt, with ethical approval number 33654.

#### 2.1.2 Chemicals and drugs

Cisplatin was supplied as vials (Oncotec Pharma Production GmbH- Germany). The contents of vial were dissolved in saline and injected intraperitoneally, at the dose 6 mg/kg once a week for 3 weeks [28].

Cyclophosphamide: (40 mg/kg, IP), three times weekly for three consecutive weeks [29]. 5-FU was purchased from S.X. Haipu Pharmaceutical Co., Ltd as ampoules (250 mg\10 ml) and rats were intraperitoneally injected by 75 mg/kg once per week for three successive weeks [30].

The kits used for the biochemical analysis were purchased from bio diagnostic Co., Cairo, Egypt. Reagents for ELIZA kit was obtained from Cloud – Clone Corp (USA). Bee honey *Nigella sativa* was obtained from the Faculty of Agriculture apiary, Cairo University, Cairo, Egypt. Diethyl nitrosamine (DEN) and CCl_4_ for induction of hepatocarcinogensis was purchased from Sigma Chemical Company (USA).

#### 2.1.3 Induction of hepatocellular carcinoma

DEN was dissolved in corn oil and intraperitoneal injected with a single dose of 50 mg/kg body weight [31]. Then two weeks later, rats were injected with a single dose of CCl_4_ (2 ml/kg IP) for carcinogenic promotion of DEN [32].

#### 2.1.4 Experimental protocol

Rats were divided into 10 groups of 15 rats each as follow:

• **Group1:** control group.
• **Group2:** normal rats orally administrated with honey, at a dose 2 g honey/rat/day [33].

**Groups3-10: rats** were IP injected with a single dose of DEN; the progress of HCC was assured histopathologically. Then, post two weeks, rats were IP injected with a single dose of carbon tetrachloride (group 4; each rat was given orally 2 g honey/rat/day as previously cited. Group 5; rats were injected with 6 mg/ kg body weight of cisplatin once a week for 3 weeks [28].Group 6; rats were medicated with cisplatin and honey was co-administered orally as previously mentioned. Group7; rats were injected with 40 mg/kg cyclophosphamide three times weekly for three weeks [29].Group 8; rats were injected with cyclophosphamide together with honey. Group 9; rats were injected IP with 75 mg/kg 5- fluorouracil once a week for three weeks, [30].Group 10; rats were medicated with 5- fluorouracil and honey).

Animals were sacrificed by decapitation post six months; the blood was withdrawn by rupture of sublingual vein after light anesthesia by diethyl ether in clean and dry test tube, left 10 min to clot and centrifuged at 3000 rpm (4 °C) for separation of serum. The separated sera were stored at −20 °C for further assessment of liver function enzymes, cholestatic biomarkers and serum total protein. Hepatic tissue was homogenized in normal physiology saline solution (0.9% NaCl) (1:9 w/v). The homogenate was centrifuged at 4 °C for 5 min at 3000 rpm. The supernatant was used for enzymes marker and antioxidant parameters determination. Hepatic lobes sections were kept in 10% formalin solution for histological examination of neoplastic nodules.

### 2.2 Methods

Serum ALT, AST [34], and ALP [35] activities were determined as biochemical markers for the early hepatic damage using quantitative colorimetric commercial kits (Biodiagnostic, ARE), whereas serum γGT was measured by the method of **Szasz** [36] using spectrum kit supplied by Egyptian Company for Biotechnology. Liver cytosolic enzyme activities GST [37], GPx [38] and GSH [39] were also detected using quantitative colorimetric kits (Biodiagnostic, ARE). Lipid peroxidation (MDA) was estimated according to Ohkawa [40]. Catalase activity was measured according to the method of **Aebi** [41], Superoxide dismutase activity was measured by the method of **Nishikimi** et al. [42], Serum alpha-fetoprotein (AFP) was determined by ELISA Biocheck kits (USA) [[43], [44], [45]]. α-L- Fucosidase (AFU) was assayed using quantitative colorimetrically kit (Biodiagnostic, ARE) [46].

### 2.3 Statistical analysis

Statistical analysis was carried out using SPSS (Version 8), one-way analysis of variance (ANOVA) computer program (mean ± SD, *n* = 15), combined with e Co-state computer program, where different letter is significant at *P* value ≤0.05.

## 3 Results

### 3.1 Effect of honey on liver functions and MDA

DEN/CCl_4_administrationshowed an increase in the activity of sera ALT, AST and ALP, in addition to MDA at (P < 0.05). After treatment with honey, a significant reduction in these parameters were observed. Cis, CY. And 5-FU treatment of DEN/CCl_4_-intoxicated rats reduced these elevated values, but the induced effects were more potent with those in case of treatment with honey plus chemotherapy. The most significant reduction was observed in G4, which was treated with honey only.

### 3.2 Effect on tissues Catalase (CAT) and superoxide dismutase (SOD) enzyme activities

DEN/CCl_4_ administration produced a significant decrease in tissues CAT and SOD activities at (P < 0.05) compared to control. Administration of honey, Cis, CY. And 5-FU individually has a significant effect on tissues CAT and SOD activities but the combination of both produced a significant increase at (P < 0.05) compared to DEN/CCl_4_ treated group. Honey and cisplatin combination (G6) have the highest significant increase at (P < 0.05), but the induced effects were less potent than those in case of treatment with honey alone (Fig. 1, Fig. 2, Fig. 3, Fig. 4, Fig. 5, Fig. 6, Fig. 7, Fig. 8, Fig. 9, Fig. 10, Fig. 11, Fig. 12, Fig. 13, Fig. 14, Fig. 15, Fig. 16, Fig. 17, Fig. 18, Fig. 19, Fig. 20, Fig. 21, Fig. 22, Fig. 23, Fig. 24, Fig. 25 ).

**Fig. 1:**
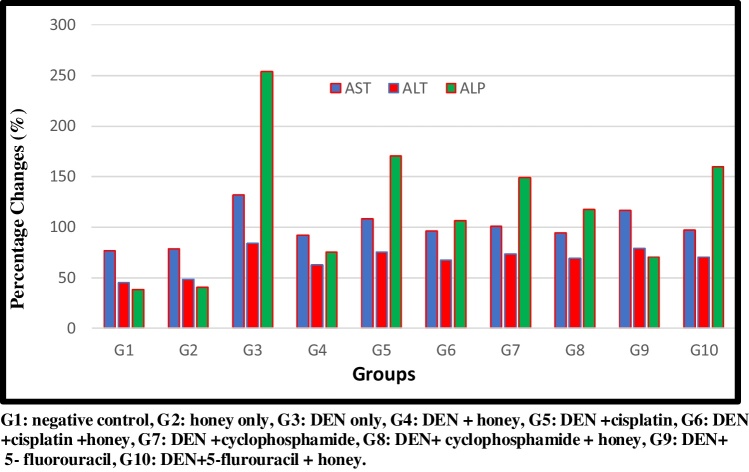
Percentage changes of AST, ALT and ALP enzyme activities in the different experimental groups. G1: negative control, G2: honey only, G3: DEN only, G4: DEN + honey, G5: DEN + cisplatin, G6: DEN + cisplatin + honey, G7: DEN + cyclophosphamide, G8: DEN + cyclophosphamide + honey, G9: DEN+ 5- fluorouracil, G10: DEN+5-flurouracil + honey.

**Fig. 2:**
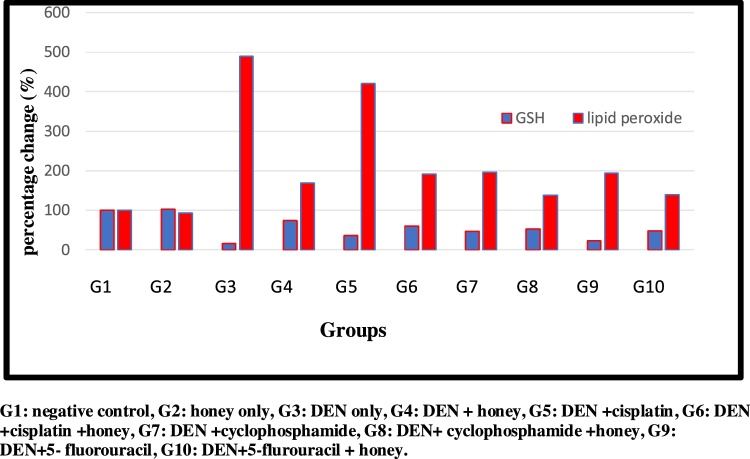
Percentage changes of GSH and Lipid peroxide levels in the different experimental groups. G1: negative control, G2: honey only, G3: DEN only, G4: DEN + honey, G5: DEN + cisplatin, G6: DEN + cisplatin + honey, G7: DEN + cyclophosphamide, G8: DEN + cyclophosphamide + honey, G9: DEN+5- fluorouracil, G10: DEN+5-flurouracil + honey.

**Fig. 3:**
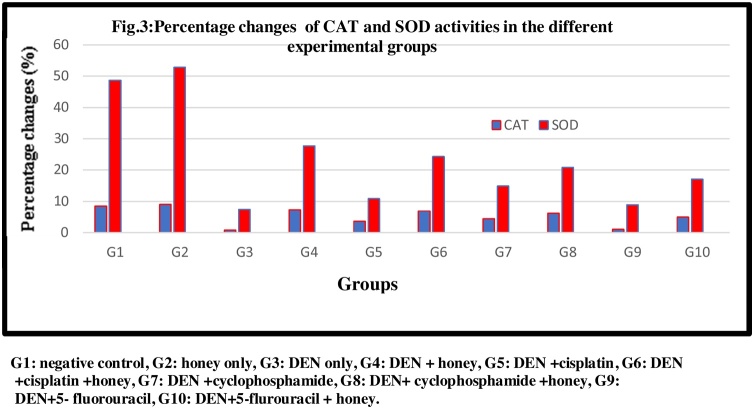
Percentage changes of CAT and SOD activities in the different experimental groups. G1: negative control, G2: honey only, G3: DEN only, G4: DEN + honey, G5: DEN + cisplatin, G6: DEN + cisplatin + honey, G7: DEN + cyclophosphamide, G8: DEN + cyclophosphamide + honey, G9: DEN+5- fluorouracil, G10: DEN+5-flurouracil + honey.

**Fig. 4:**
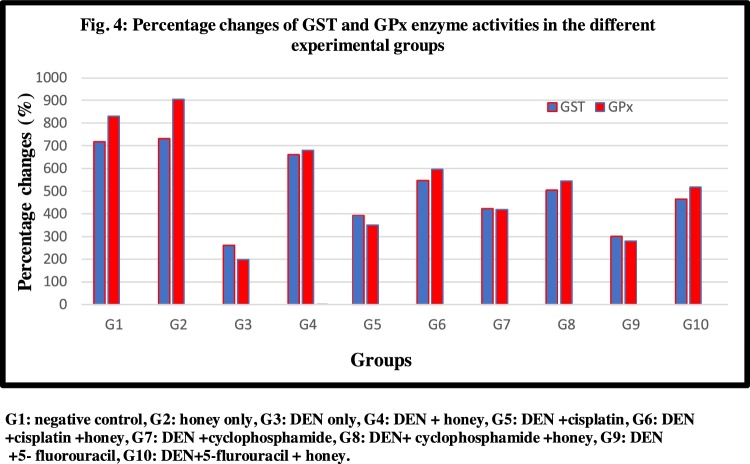
Percentage changes of GST and GPx enzyme activities in the different experimental groups. G1: negative control, G2: honey only, G3: DEN only, G4: DEN + honey, G5: DEN + cisplatin, G6: DEN + cisplatin + honey, G7: DEN + cyclophosphamide, G8: DEN + cyclophosphamide + honey, G9: DEN +5- fluorouracil, G10: DEN+5-flurouracil + honey.

**Fig. 5:**
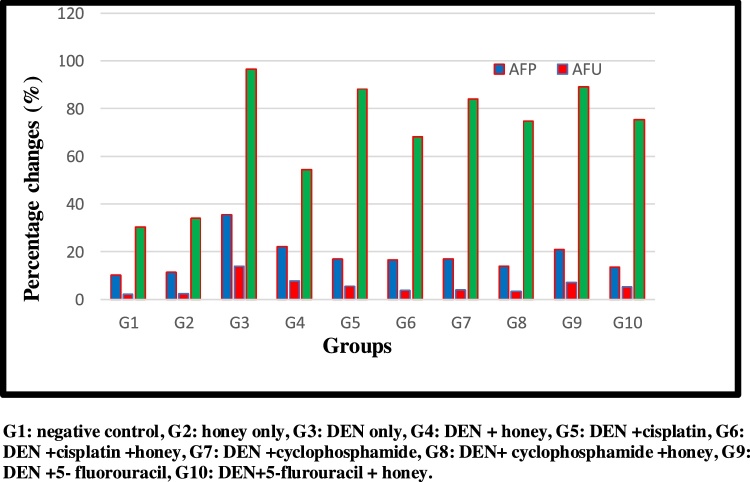
Percentage changes of AFP. AFU and GGT in the different experimental groups. G1: negative control, G2: honey only, G3: DEN only, G4: DEN + honey, G5: DEN + cisplatin, G6: DEN + cisplatin + honey, G7: DEN + cyclophosphamide, G8: DEN + cyclophosphamide + honey, G9: DEN +5- fluorouracil, G10: DEN+5-flurouracil + honey. Histopathological examination:

**Fig. 6:**
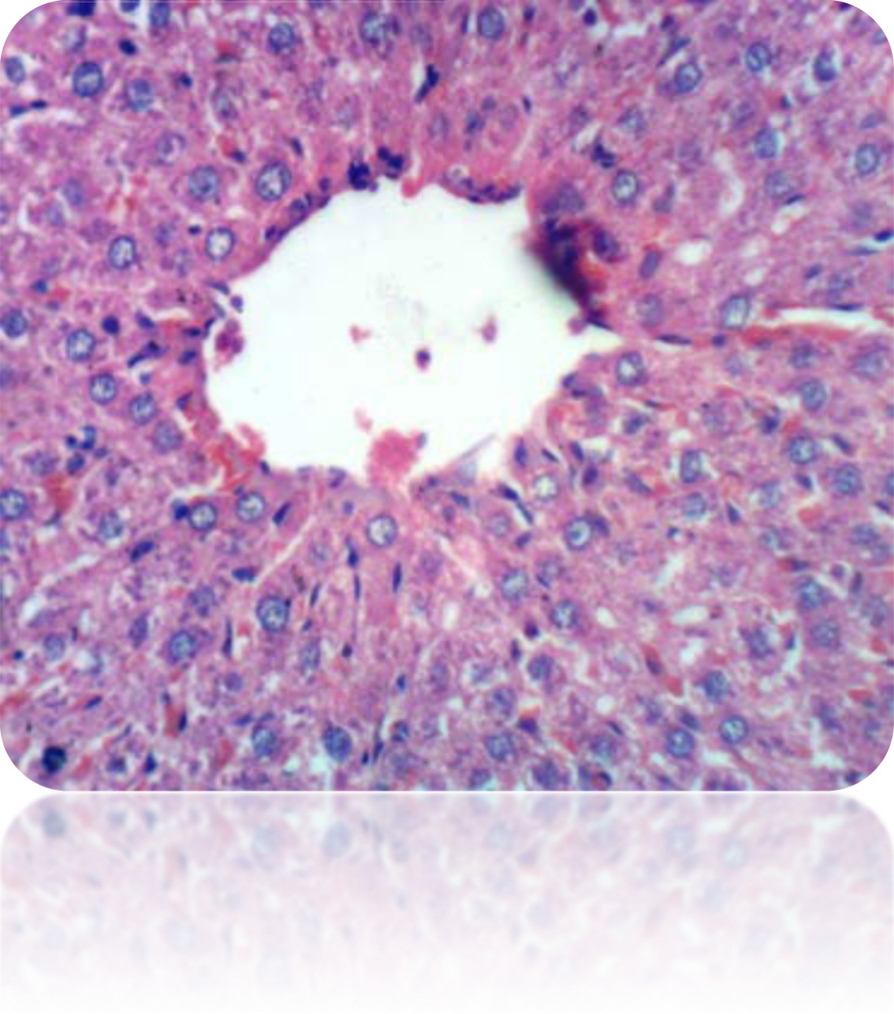
Liver of rat from control group showed normal histological structure of hepatic lobule (H & E X 400).

**Fig. 7:**
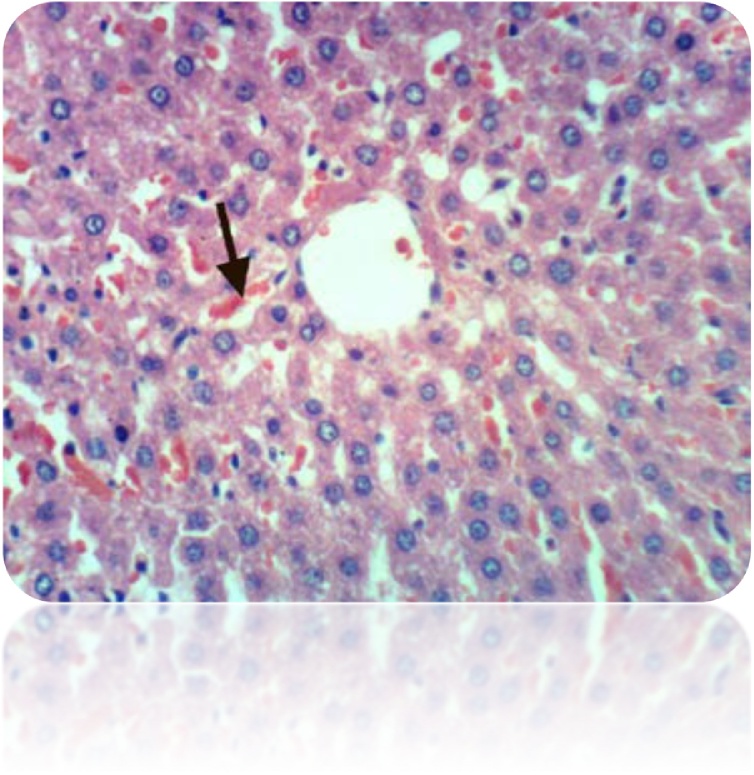
Liver of rat from honey administrated group showed slight congestion of hepatic sinusoids (H & E X 400).

**Fig. 8:**
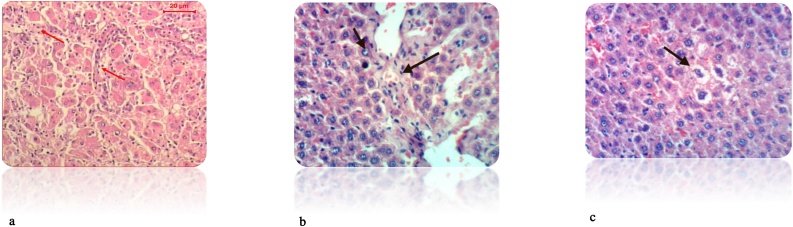
(a): liver section of DEN-treated rat showed many of the well differentiated tumor cells, they are arranged in cords like pattern (red arrows) (H& E stain, Scale Bar: 20 μm). (b): Liver of rat from DEN/CCl_4_ intoxicated group showed karyomegaly of hepatocytic nuclei and fine strands of collagen fibers deposition (H & E X 400). (c): Liver of rat from DEN/CCl_4_ intoxicated group showed clear cell foci of hepatocytes (H & E X 400).

**Fig. 9:**
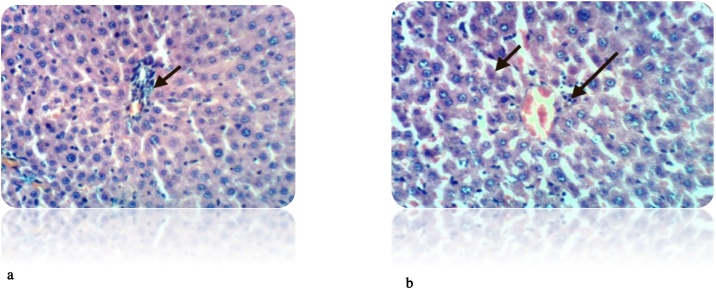
(a): Liver of rat from DEN/CCl_4_ intoxicated group treated by honey showed proliferation of oval cells (H & E X 400). (b): Liver of rat from DEN/CCl_4_ intoxicated group treated by honey showed necrosis of sporadic hepatocytes and proliferation of oval cells (H & E X 400).

**Fig. 10:**
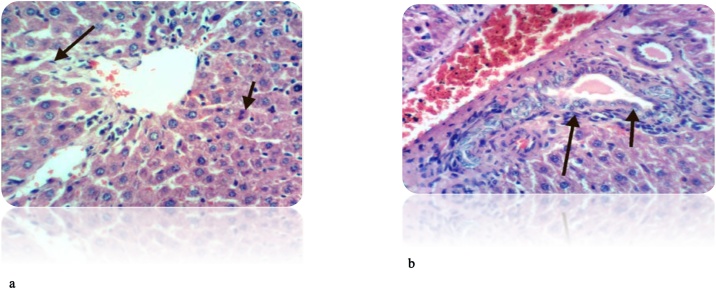
(a): Liver of rat from DEN/CCl_4_ intoxicated group treated by cisplatin showed necrosis of sporadic hepatocytes and fine strands of collagen fibers deposition (H & E X 400). (b): Liver of rat from DEN/CCl_4_ intoxicated group treated by cisplatin showed hyperplasia of epithelial lining bile duct and fibroplasia in portal triad(H & E X 400).

**Fig. 11:**
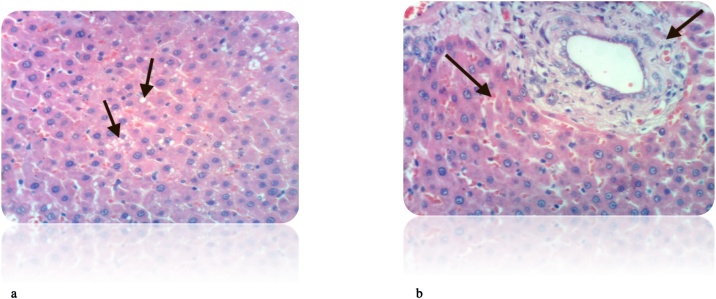
(a): Liver of rat from DEN/CCl_4_ intoxicated group treated by cisplatin and honey showed cytoplasmic vacuolization of hepatocytes (H & E X 400). (b): Liver of rat from DEN/CCl_4_ intoxicated group treated by cisplatin and honey showed fibroplasia, collagen fibers deposition in the portal triad and congestion of hepatic sinusoids (H&E X 400).

**Fig. 12:**
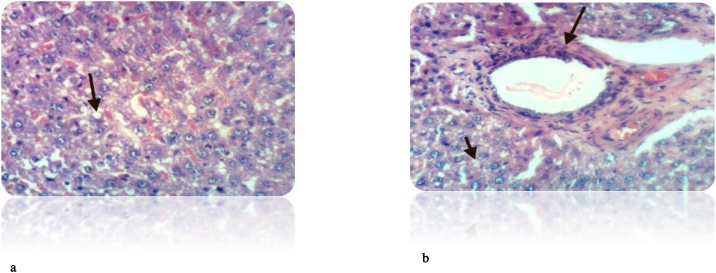
(a): Liver of rat from DEN/CCl_4_ intoxicated group treated by cyclophosphamide showed cytoplasmic vacuolization of hepatocytes (H & E X 400). (b): Liver of rat from DEN/CCl_4_ intoxicated group treated by cyclophosphamide showed fibroplasia and collagen fibers deposition in portal triad as well as cytoplasmic vacuolization of hepatocytes (H & E X 400).

**Fig. 13:**
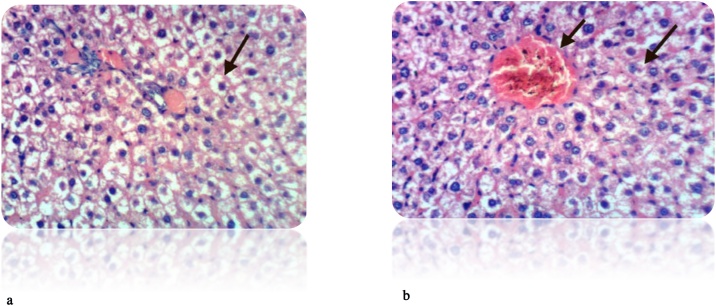
(a): Liver of rat from DEN/CCl_4_ intoxicated group treated by cyclophosphamide and honey showed hydropic degeneration of hepatocytes (H & E X 400). (b): Liver of rat from DEN/CCl_4_ intoxicated group treated by cyclophosphamide and honey showed congestion of central vein and hydropic degeneration of hepatocytes (H & E X 400).

**Fig. 14:**
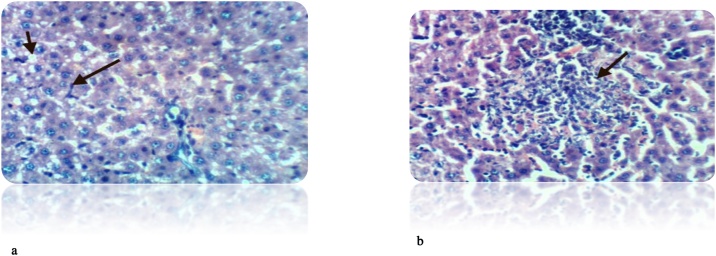
a): Liver of rat from DEN/CCl_4_ intoxicated group treated by 5-FU showed cytoplasmic vacuolization of hepatocytes and Kupffer cells activation (H & E X 400). (b): Liver of rat from DEN/CCl_4_ intoxicated group treated by 5-FU showed focal hepatic necrosis associated with inflammatory infiltration (H & E X 400).

**Fig. 15:**
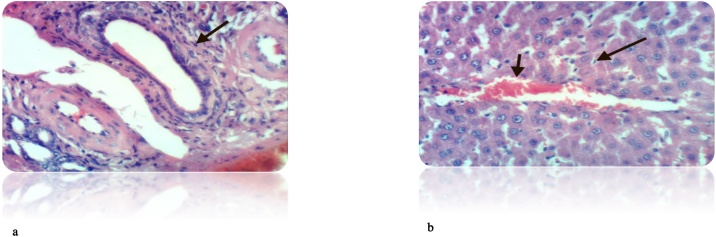
a): Liver of rat from DEN/CCl_4_ intoxicated group treated by 5-FU and honey showed fibroplasia and collagen fibers deposition in the portal triad (H & E X 400). (b): Liver of rat from DEN/CCl4 intoxicated group treated by 5-FU and honey showed congestion of central vein and Kupffer cells activation (H & E X 400). Histochemical reaction for collagen fiber:

**Fig. 16:**
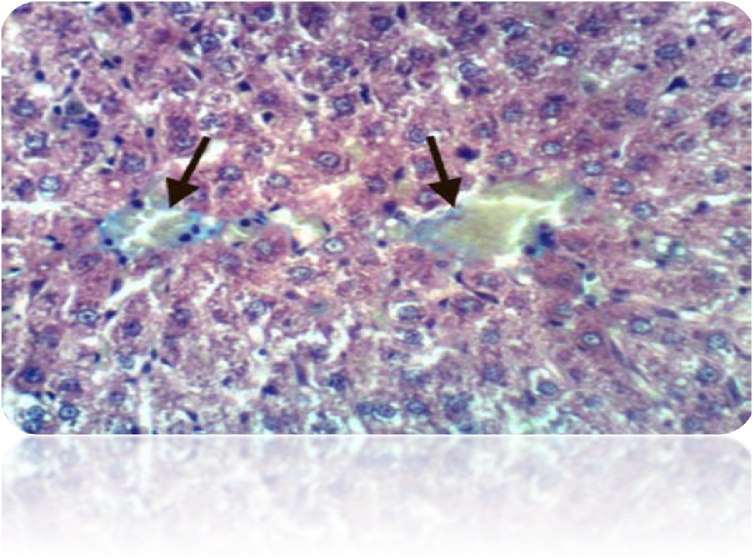
Liver of rat from control group 1 showed no histochemical reaction for collagen fibers (Masson’s Trichrome Stain X 400).

**Fig. 17:**
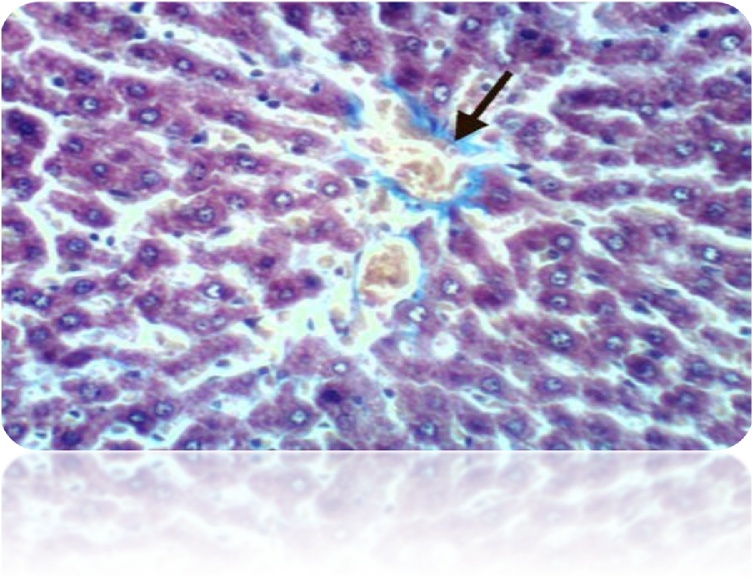
Liver of rat from honey administrated group showed no histochemical reaction for collagen fibers (Masson’s Trichrome Stain X 400).

**Fig. 18:**
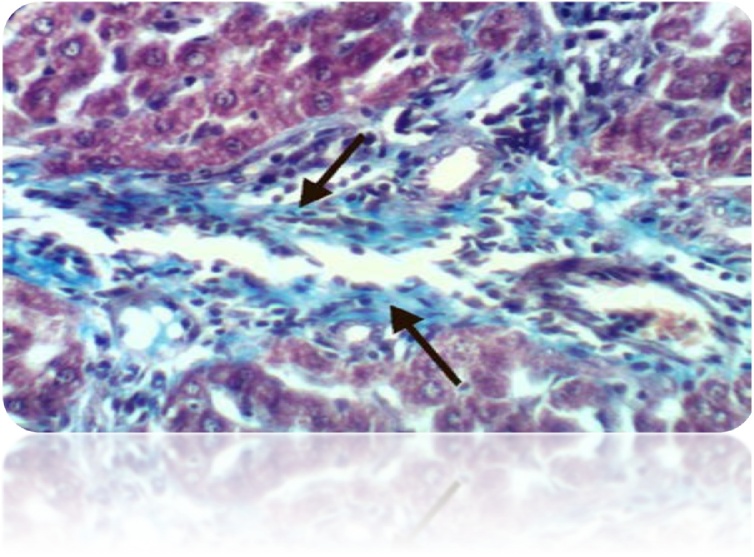
Liver of rat from DEN/CCl_4_ intoxicated group showed strong positive histochemical reaction for collagen fibers (Masson’s Trichrome Stain X 400).

**Fig. 19:**
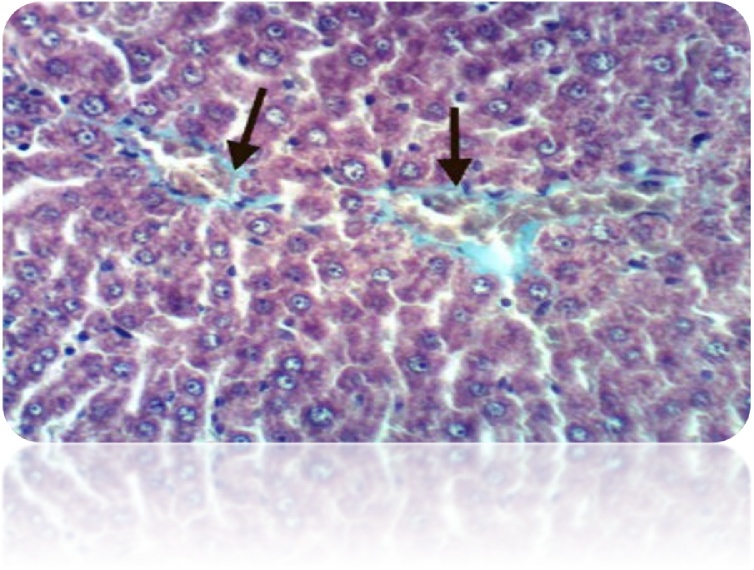
Liver of rat from DEN/CCl_4_ intoxicated group treated by honey showed no histochemical reaction for collagen fibers (Masson’s Trichrome Stain X 400).

**Fig. 20:**
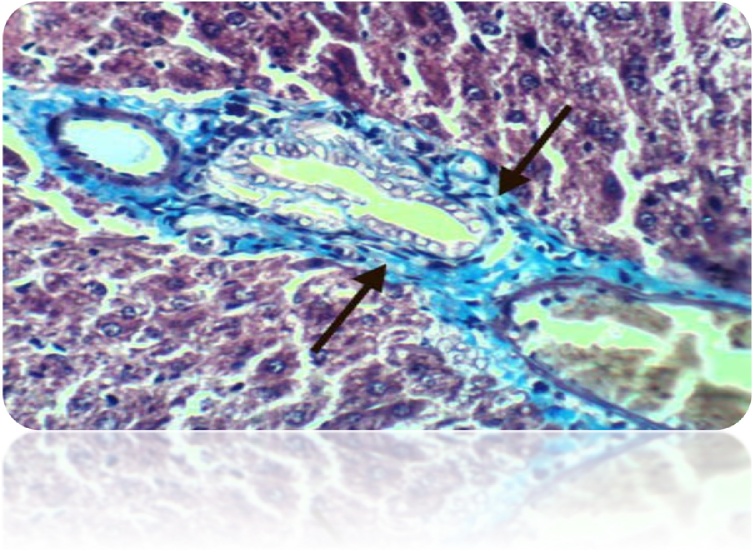
Liver of rat from DEN/CCl_4_ intoxicated group treated by cisplatin showed positive histochemical reaction for collagen fibers (Masson’s Trichrome Stain X 400).

**Fig. 21:**
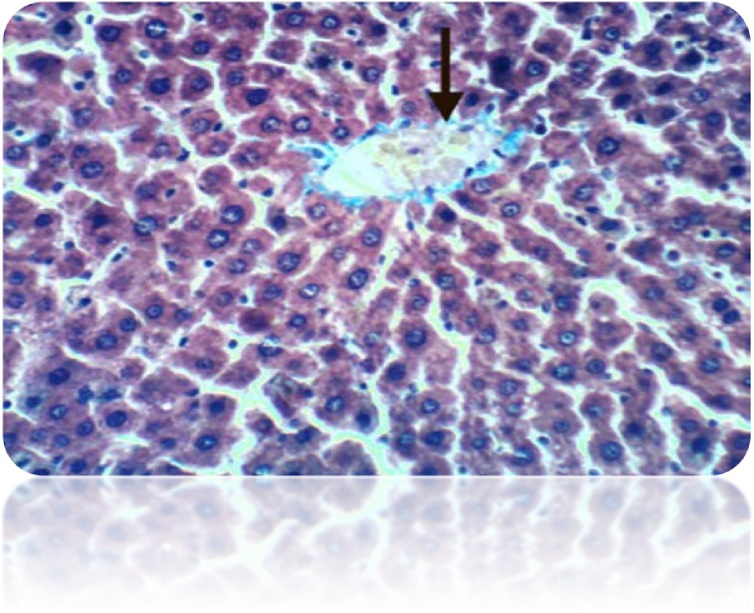
Liver of rat from DEN/CCl_4_ intoxicated group treated by cisplatin and honey showed no histochemical reaction for collagen fibers (Masson’s Trichrome Stain X 400).

**Fig. 22:**
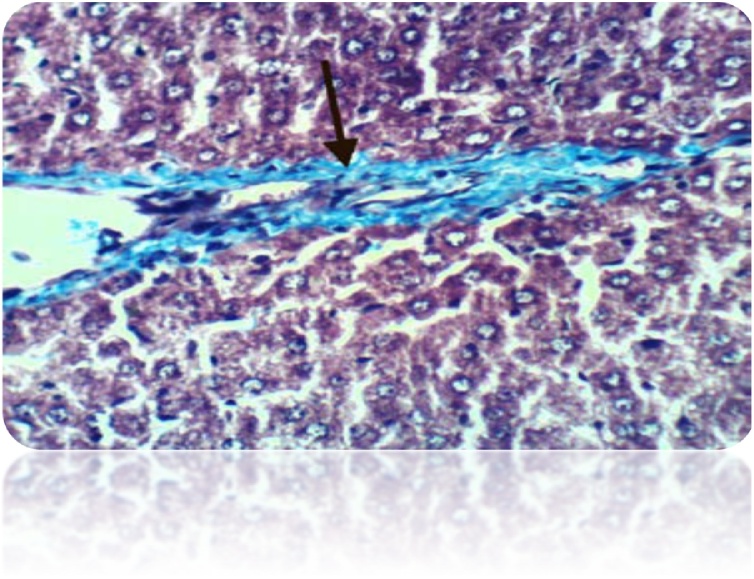
Liver of rat from DEN/CCl_4_ intoxicated group treated by cyclophosphamide showed positive histochemical reaction for collagen fibers (Masson’s Trichrome Stain X 400).

**Fig. 23:**
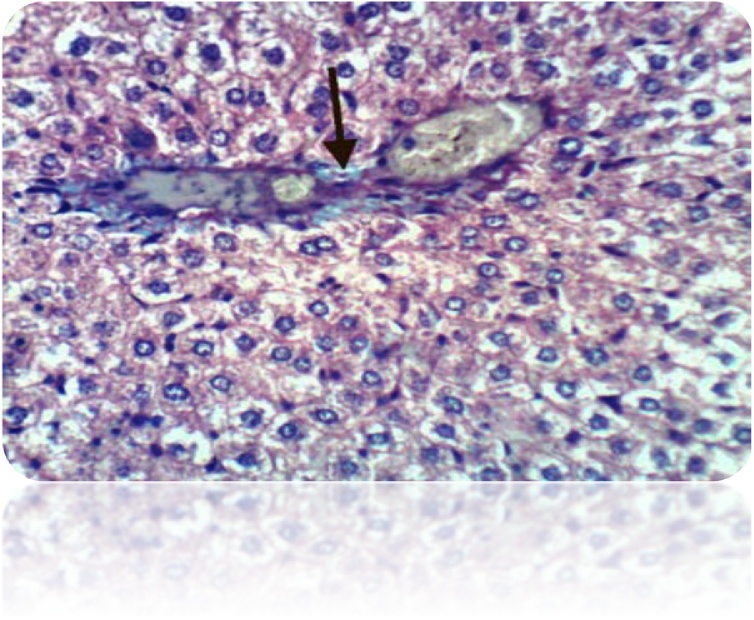
Liver of rat from DEN/CCl_4_ intoxicated group treated by cyclophosphamide and honey showed no histochemical reaction for collagen fibers (Masson’s Trichrome Stain X 400).

**Fig. 24:**
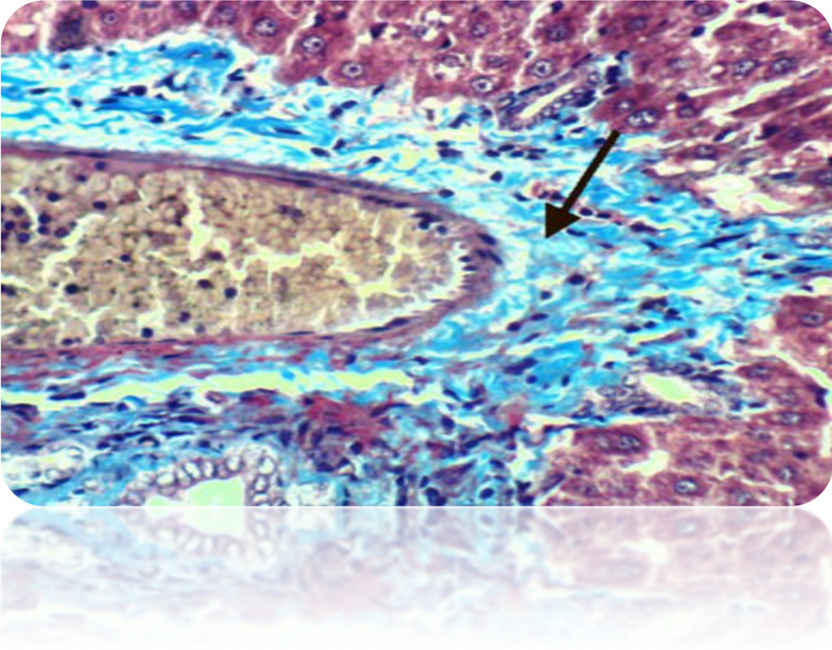
Liver of rat from DEN/CCl_4_ intoxicated group treated by 5-FU showed strong positive histochemical reaction for collagen fibers (Masson’s Trichrome Stain X 400).

**Fig. 25:**
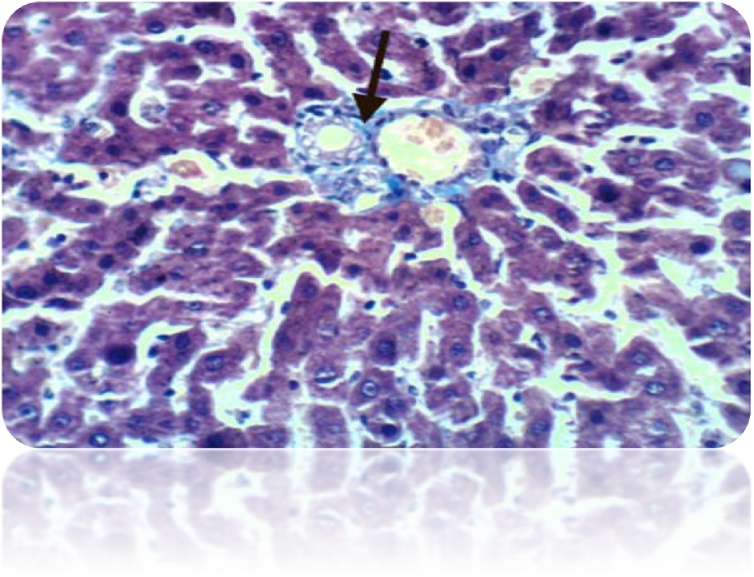
Liver of rat from DEN/CCl4 intoxicated group treated by 5-FU and honey showed no histochemical reaction for collagen fibers (Masson’s Trichrome Stain X 400).

### 3.3 Effect on tissues Glutathione peroxidase (GPx), glutathione-s-transferase (GST) and Glutathione (GSH)

Tissues levels GP_x_, GST and GSH in DEN/CCl_4_- intoxicated rats showed a significant reduction than normal control (P < 0.05), after treatment of DEN/CCl_4_- intoxicated rats with honey, a significant elevation was observed (P < 0.05) and the best enhancement was observed in honey plus cisplatin rats. As well bee honey still the most effective one.

### 3.4 Hepatocellular carcinoma GGT, AFP and AFU

Serum AFP level of DEN/CCl_4_-intoxicated rats was significantly increased, compared to normal (P < 0.05). After treatment with 5-FU, a significant reduction in serum AFP was observed at (P < 0.05), the reduction by the combination of 5-FU and honey was more pronounced. In case of AFU, the addition of honey to CY is the more pronounced one. Honey alone diminishes the elevation of GGT.

## 4 Discussion

No single or combination chemotherapy regimen has been found to be specifically effective in hepatocellular carcinoma, despite the great numbers of forced and passivity studies have been performed with most classes of chemotherapeutic agents [47]. Diethyl nitrosamine (DEN) is a well-known hepatocarcinogenic agent used in the experimental animals [48]. In the present study, while treatment with DEN/CCl_4_was effective in inducing HCC in rats, bee honey, administered either alone or in combination with different chemotherapeutic agents was effective in ameliorating the hepato-carcinogenic effect of DEN/CCl_4_.

Table 1 demonstrates the effectiveness of DEN/CCl4 in inducing liver dysfunction measured as elevated AST, and ALT compared to control group. This elevation which is due to leakage from damaged or necrotic cells can find supports in multiple studies and can be used as evidence for HCC development in rats intoxicated with DEN [49]. Table 1 also demonstrates the ameliorating effects of the used drugs either independently or in combination with honey. Cyclophosphamide was the more effective followed by 5-FU, and then cisplatin. The synergistic effect of honey can be easily observed as much lower decrease of AST and ALT in groups of animals co-administered drugs/honey (G6, G8, and G10).

**Table 1:** Activities of AST, ALT, ALP enzymes and MDA level in the different experimental groups. Results are given in mean ± SD from 15 rats in each group. Values in parentheses represent % of improvements from control.

Groups Parameters	G1	G2	G3	G4	G5	G6	G7	G8	G9	G10
AST (U/l)	76.6 ± 4.97^f^	78.8 ± 7.08^f^	131.8 ± 4.96^a^	92.2 ± 3.11^e (51.69)^	108.6 ± 4.72^c (30.28)^	96.4 ± 3.57^d,e (46.21)^	100.6 ± 2.70^d (40.73)^	94.4 ± 2.40^d,e (48.83)^	116.6 ± 2.70^b (19.84)^	97.2 ± 1.48^d,e (45.16)^
ALT (U/l)	45.2 ± 4.14 ^e^	48.6 ± 7.23 ^e^	84.2 ± 3.49 ^a^	63.00 ± 2.00^d (46.90)^	75.6 ± 5.12 ^a,b,c (19.02)^	67.2 ± 5.01^c, d (37.61)^	73.8 ± 5.54 ^b,c (23.00)^	69 ± 7.38^c,d (33.62)^	79.00 ± 9.98^a,b (11.50)^	70.6 ± 4.82^c,d (30.08)^
ALP	38.49 ± 2.34^g^	40.52 ± 2.08 ^g^	253.51 ± 19.53 ^a^	75.67 ± 7.03^f (462.04)^	170.16 ± 13.46^b,c (216.54)^	106.72 ± 8.72^e (381.37)^	148.80 ± 16.89^d (272.04)^	117.29 ± 6.87^e (353.91)^	183.81 ± 6.25 ^b (181.08)^	160.00 ± 12.54^c,d (242.94)^
MDA (nmol MDA / g. tissue)	8.41 ± 0.85^g^	7.88 ± 1.05^g^	41.21 ± 1.52^a^	14.25 ± 1.68^e (320.57)^	35.37 ± 0.77^b (69.44)^	16.08 ± 0.52^d (298.81)^	16.50 ± 1.40^d (293.81)^	11.66 ± 1.05^f (351.36)^	24.73 ± 2.59^c (195.95)^	11.72 ± 0.89^f (350.65)^

G1: negative control, G2: honey only, G3: DEN + CCL_4_, G4: DEN + CCL_4+_Honey, G5: DEN + CCL_4_+cisplatin, G6:DEN + CCL_4_+cisplatin + honey,G7:DEN + CCL_4_+cyclophosphamide, G8: DEN + CCL_4_+cyclophosphamide + honey, G9: DEN + CCL_4_+5-flurouracil, G10: DEN + CCL_4_+5-flurouracil + honey.

Statistical analysis are carried out using one way analysis of variance (ANOVA) using Co-Stat Computer program, where unshared letters are significant at *p≤*0.05.

On the other hand, the remarkable increase of ALP as marker of DEN/ CCl_4_ liver toxicity (G3) can be attributed to a mechanical obstruction of bile ducts, failure to excrete the enzyme, and thus its increase in the blood [50]. Reduction of ALP activity post treatment with the three studied drugs either independently or in combination with honey is also presented in Table 1 or might be attributed to the decrease of mechanical obstruction in the bile duct. The hepatoprotective effect of honey reported in the present study is in good agreement with the previous work of **Yaman** et al. [51] who reported a hepatoprotective effect of honey against aflatoxin induced hepatic damage.

Lipid peroxides measured as MDA is broadly utilized as one of the most important indices of oxidative stress to evaluate the oxidative damage in patients with liver injury [[52], [53], [54]]. The oxidative toxic effect of DEN/CCl4 can easily been observed in Table 2 as significant increase of MDA together with significant decrease of GSH as non-enzymatic antioxidant, catalase, GPx, SOD, and GST as major antioxidant enzymes critically needed for the scavenging of MDA as marker of oxidative stress, compared to controls. This can find good support in the recent study of DEN/CCl4 **Zhang** et al. [55] who reported that DEN via interaction with strategic macromolecules such as antioxidant enzymes, DNA, lipids, and DNA repairing system enzymes can induce HCC. Moreover, it is well accepted that CCl4 biotransformation by the help cytochrome P-450 usually converted to trichloromethyl free radical (CCl_3_*), and trichloromethyl proxy free radical (CCl_3_OO*) as two metabolites related to ROS generation, lipid peroxidation, and decrease of CAT, SOD, GST, and GPx enzymatic activities [[56], [57], [58]].Moreover, the reported data are in concomitant with the previous study of Hussein & and Khalifa and Kadasa et al. [59,60] who indicated significant reduction of antioxidant enzyme activities and their relative gene expression in DEN-induced rats comparing to control.

**Table 2:** Antioxidants CAT, SOD, GPx, GST enzymes and liver contents of GSH in different experimental groups. Results are given in mean ± SD from 15 rats in each group. Values in parentheses represent % of improvements from control.

	G1	G2	G3	G4	G5	G6	G7	G8	G9	G10
CAT (μMol/min / mg protein)	8.48 ± 0.05 ^b^	8.94 ± 0.09 ^a^	0.80 ± 0.05 ^j^	7.24 ± 0.45 ^c (75.94)^	3.64 ± 0.11 ^h (33.49)^	6.95 ± 0.06 ^d (72.52)^	4.43 ± 0.07 ^g (42.80)^	6.16 ± 0.08 ^e (63.20)^	1.04 ± 0.05 ^i (2.83)^	4.90 ± 0.06 ^f (48.34)^
SOD (μMol /min / mg protein)	48.61 ± 0.86 ^b^	52.79 ± 2.36 ^a^	7.42 ± 0.29 ^j^	27.77 ± 0.53 ^c (41.86)^	10.81 ± 0.33^h (6.97)^	24.35 ± 1.36 ^d (34.82)^	14.94 ± 0.50 ^g (15.47)^	20.75 ± 0.47 ^e (27.42)^	8.89 ± 0.42 ^i (3.02)^	17.05 ± 0.33 ^f (19.81)^
GPx (n Mol /min /mg protein)	830.46 ± 30.30^b^	906 ± 20.98^a^	197.9 ± 37.18^i^	680.80 ± 44.11^c (58.14)^	349.96 ± 38.24^g (18.3)^	594.96 ± 24.96^d (47.81)^	419.97 ± 37.26^f (26.74)^	544.62 ± 22.75^e (41.75)^	280.06 ± 36.05^h (9.89)^	517.30 ± 32.01^e (38.46)^
GST (nmol /min /mg protein)	717.40 ± 3.21^b^	731.34 ± 4.08^a^	260.7 ± 4.24^j^	660.81 ± 6.96^c (55.76)^	393.2 ± 3.60^h (18.46)^	546.64 ± 5.21^d (39.85)^	423.37 ± 5.06^g (22.67)^	505.03 ± 3.73^e (34.05)^	299.66 ± 7.60^i (5.42)^	464.98 ± 4.74^f (28.47)^
GSH (m Mol/g)	1327.31 ± 6.88^b^	1365.84 ± 5.11^a^	212.64 ± 19.26^j^	975.36 ± 8.71^c (57.46)^	477.26 ± 7.77^h (19.93)^	790.31 ± 6.42^d (43.52)^	633.92 ± 18.60^g (31.73)^	711.92 ± 11.61^e (37.61)^	299.95 ± 6.12^i (6.57)^	649.53 ± 8.55^f (32.92)^

G1: negative control, G2: honey only, G3: DEN + CCL_4_, G4: DEN + CCL_4_+Honey, G5: DEN + CCL_4_+cisplatin, G6:DEN + CCL_4_+cisplatin + honey,G7:DEN + CCL_4_+cyclophosphamide,G8:DEN + CCL_4_+cyclophosphamide + honey, G9: DEN + CCL_4_+5-flurouracil, G10: DEN + CCL_4_+5-flurouracil + honey.

Statistical analysis are carried out using one way analysis of variance (ANOVA) using Co-Stat Computer program, where unshared letters are significant at *p≤*0.05.

The current study also showed a significant increase in the antioxidant enzymes (CAT, SOD, GST, and GPx) after administration of honey to healthy group (G2). In addition, administration of honey to DEN/CCl_4_ intoxicated rats (G4) either alone or with other chemotherapeutic drugs (G6, G8, and G10) demonstrated an elevation in these antioxidant enzymes compared to the groups treated with chemotherapy only (G5, G7 and G9). This observation may be attributed to the antioxidant property of honey because of the presence of phenolic compounds [61]. Phenolic compounds have hydroxyl groups connected to the aromatic ring that can act as hydrogen donors in scavenging of free radicals [62]. Also, phenolic compounds are electron donor and can reduce the metal ions. So, it has been believed that the phenolic content holds the key to antioxidant property of the honey [51,63].

The independent therapeutic effects of cisplatin, cyclophosphamide, and 5FU, reported in the present study are in good agreement with multiple studies which prove the antioxidant, anti-cancer, and anti-inflammatory effects of these drugs [[64], [65], [66]].

It was proven that ROS are a direct cause of somatic cell mutagenesis and they are cancer promoter [67], so they are considered as life threatening and oncogenes product. On the other hand, the production of ROS is characteristic feature for all chemotherapeutic drugs due to their abilities to provoke malignant cell death [68]. These points to the different roles of ROS in different stages of tumor development and death [69].

Alpha-fetoprotein (AFP) is a fetal glycoprotein associated with tumor. During the early stage of hepatocarcinogensis, reactivation of AFP gene is detected in the hepatocyte’s liver; cytoplasmic AFP enhances the proliferation of malignant liver cells. Also, the extracellular AFP accelerates the growth of malignant hepatocytes which is mediated by AFP receptor [70]. Along with hepatocytes, liver progenitor cells (LPC) also develop AFP during their cellular differentiation [71]. The LPCs play a critical role in liver homeostasis and regeneration [72,73]. Elevation of serum AFP is indicative of the proliferation of LPC as a response to chronic liver injury or HCC development [74].

Our study showed significant elevation in AFP level after injection with DEN/CCl_4_ compared to normal untreated control rats. This observed elevation in AFP is an indicative to not only the hepatic damage but also, the development of HCC. This result agrees with the previous studies of **Kadasa** et al. [60]**; Zhang** et al. [55], who reported the elevation in AFP level in DEN intoxicated rats compared to normal rats. The elevated level indicated the carcinogenic effect of DEN and induction of HCC, as AFP is used to differentiate between HCC and chronic liver diseases. In addition, **Hussain** et al. [75], found that during the metabolic biotransformation of DEN, pro mutagenic products are produced which are responsible for the carcinogenic effect of DEN. Hence, activation of AFP gene and elevation in its serum level (Table 3, Table 4).

**Table 3:** GGT enzyme activity and AFP, AFU inflammatory markers in the different experimental groups. Results are given in mean ± SD from 15 rats in each group. Values in parentheses represent % of improvements from control.

	G1	G2	G3		G4	G5	G6	G7	G8	G9	G10
**GGT**	30.33 ± 2.99^h^	34.02 ± 3.96^h^	96.57 ± 3.12^a^		54.40 ± 3.20^g (139.03**)**^	88.23 ± 2.50^c (27.49)^	68.31 ± 3.17^f (93.17**)**^	84.05 ± 2.10^d (41.27**)**^	74.80 ± 3.12^e (71.77**)**^	89.17 ± 2.78^c (24.39**)**^	75.49 ± 2.99^e (69.50**)**^
**AFP**	10.10 ± 0.01^d^	11.34 ± 0.97^d^		35.68 ± 1.83^a^	22.16 ± 0.51^b (133.86**)**^	17.02 ± 1.33^c (184.75)^	16.51 ± 0.87^c (189.80**)**^	17.02 ± 1.33^c (184.75**)**^	14.09 ± 0.81^c (213.76)^	21.02 ± 4.11^b (145.148**)**^	13.64 ± 0.64^c^(218.21)
**AFU**	2.18 **± 0.21^e^**	2.35 ± 0.41^e^	13.96 ± 1.87^a^		7.87 ± 0.83^b (279.35**)**^	5.52 ± 0.19^c (387.15**)**^	3.73 ± 0.30^d (469.22**)**^	3.97 ± 0.32^d 458.25))^	3.31 ± 0.20^d (488.53**)**^	7.25 ± 0.43^b (307.79**)**^	5.11 ± 0.38^c^(405.96)

G1: negative control, G2: honey only, G3: DEN + CCL4, G4: DEN + CCL4+Honey, G5: DEN + CCL4+cisplatin, G6:DEN + CCL4+cisplatin + honey,G7:DEN + CCL4+cyclophosphamide, G8: DEN + CCL4+cyclophosphamide + honey, G9: DEN + CCL4 + 5-flurouracil, G10: DEN + CCL4 + 5-flurouracil + honey.

Statistical analysis are carried out using one way analysis of variance (ANOVA) using Co-Stat Computer program, where unshared letters are significant at p ≤ 0.05.

**Table 4:** Lesion score of histopathological examination in different treated group.

Histopatholo-gical lesion	control	honey	DEN	DEN/CCl_4_	DEN/CCl_4 + honey_	DEN/CCl_4 + cisplatin_	DEN/CCl_4 + Cisplatin + honey_	DEN/CCl_4 + Cyclo_	DEN/CCl_4 + Cyclo + honey_	DEN/CCl_4 + 5-FU_	DEN/CCl_4 + 5-FU + honey_
well differentiated tumor cells (HCC)	0	0	2	2	0	0	0	0	0	0	0
karyomegaly of hepatocytic nuclei	0	0	3	3	2	1	1	2	0	1	0
clear cell foci of hepatocytes	0	0	1	2	1	1	0	1	0	0	0
proliferation of oval cells	0	0	3	3	2	2	1	2	1	2	1
necrosis of sporadic hepatocytes	0	0	3	3	3	2	2	2	1	1	1
fibroplasia in portal triad	0	0	2	3	2	2	2	2	1	2	2
cytoplasmic vacuolization of hepatocytes	0	0	3	3	2	1	1	3	3	2	1
focal hepatic necrosis associated with inflammatory infiltration	0	0	2	3	2	1	1	2	0	2	0
Mean ± SD Percentages Changes			6.33 ± 0.22^a^	7.33 ± 0.12^b + 15.79^	4.66 ± 0.05^c −36.43^	3.33 ± 0.02^d −54.70^	2.66 ± 0.01^e -63.71^	4.70 ± 0.03^c −35.87^	2.00 ± 0.02^f −72.71^	3.33 ± 0.04^d -54.70^	1.66 ± 0.01^f −77.35^

Biomarkers histopathological examination revealed significant reduction in DEN/CCl4 group treated by honey, cisplatin and cisplatin with honey demonstrated the remarkable reduction in HCC group treated with cisplatin and honey with percentage reached to -63.71%. However, the percentages reduction in the parameters of histopathological examination showed more sever reduction in HCC group treated with cyclophosphamide.

Besides, treatment of intoxicated rats with cisplatin showed significant decrease in AFP level compared to DEN-intoxicated rats. This may be attributed to the anticancer effect of cisplatin. AFP is indicative for HCC; the decrease in its level suggested the inhibition in HCC development which is also supported by the improvement of liver function enzymes activity compared to HCC bearing rats. Our results are in concomitant also with **Abdel-Hamid** et al. [76], who reported a significant decrease in AFP level compared to rats injected with sub carcinogenic dose of DEN, which reflected the response to cisplatin effect. Previously, **Keam *et al.*** [77], observed the fall off in AFP level after cisplatin treatment and suggested that patients with HCC who did not show tumor response to radiographic treatment may response to cisplatin treatment.

The anticancer effects of cisplatin, cyclophosphamide, and 5FU as chemotherapeutic drug used in the present study can easily related to the remarkable decrease of AFP as marker of HCC. This can be supported through related studies which found a decrease in AFP level after using these three drugs [[77], [78], [79], [80]].

Administration of honey either alone or with other anticancer drugs markedly decreased the AFP level compared to rats treated with drugs alone which prove its synergistic effect. The reported anticancer effect of honey may result from inhibition of DNA synthesis or down regulation of MMP-2 and MMP-9, which are involved in the induction of angiogenesis process, apoptotic and cytotoxic effects [81,82].

Alpha-l-fucosidase (AFU) is studied as one of the better generally used HCC marker as many researches indicated its considerable elevation in HCC patients correlated to patients with benign liver diseases [[83], [84], [85], [86], [87]]. The current study demonstrated significant altitude in AFU activity in DEN/CCl_4_ intoxicated rats correlated to normal untreated one. analogous effect was found by **Abdallah and Khattab** [88], who found elevation in AFU enzyme activity in both cytosol and serum in DEN-treated rats as compared to normal one. In a parallel result with **El- Attwa** et al. [89] who found a significant elevation in AFU level which is correlated well with the tumor size. This may be connected to the growing in protein synthesis in the tumor cells with an ensuing elevation in fucose turnover [90]. **Zahran** et al. [91], found an increase in AFU level in DEN-treated rats. DEN is metabolized to active ethyl radical metabolites that react with DNA performing in mutation followed by carcinogenesis [92]. **Also, Moriwaki** et al. [93] reported that during the hepatocarcinogensis process, fucosylation of sugar proteins are elevated, thus leading to an increase in AFU enzyme activity. **Gan** et al. [94], suggested that AFU enzyme activity is corresponded with the tumor growth and its contraction is refer to chemotherapeutic response. These results backed the hepatocarcinogenic effect of DEN. Also, **Dai** et al. [95]; **Chen** et al. [96]; **Ahmed** et al. [97]; **Shahat** et al. [98], supported our results.

Treatment of DEN/CCl_4_ intoxicated rats with the various chemotherapeutic drugs display a significant decrease in AFU level that may be attributed to the success of these drugs to inhibit the tumor propagation as an anticancer drug. In addition, **Montaser** et al. [99] **and Hassan** et al. [100] also supported our results.

Honey supplementation either alone or with the chemotherapeutic drugs showed higher percentage of improvement than that treated with drugs alone. This may be correlated with its composition as it contains lipids, carotenoids, anthraquinones, organic acids and flavonoids that are proven to have anticancer effect. In a parallel with our results, **Hussein and Khalifa** [59], found that treatment of HCC bearing animals with Ellagitannin flavonoids caused significant depletion in AFU compared to DEN-intoxicated rats, reflecting the effect of flavonoids as anticancer. Our result was confirmed by the studies of **Shaker** et al. [101]; **Ahmed** et al. [97]; **Hamza** et al. [102].

Regarding to, gamma-glutamyl transferase (GGT), it is a glycoprotein enzyme, that is located on the cell membranes of most body tissues, but it is more commonly found in hepatocytes, and is routinely used as biomarker for liver injury and excessive alcohol consumption [[103], [104], [105]]. The main function of GGT is the extracellular catabolism of glutathione which cause production of ROS [106,107].

Glutathione plays a critical role in protecting cells against the resultant oxidants during normal metabolism. The reaction in which GGT catalyzes is the transfer of a glutamyl residue to an acceptor, helping in maintaining adequate glutathione levels. Moreover, GGT is also involved in the metabolism of leukotriene and movement of amino acids across the cell membrane [105,108]. The blockage of bile ducts or liver damage can cause accumulation of GGT in the liver and excess secretion of GGT into the blood. As a result, the elevation of GGT level in serum can be indicative for potential hepatic or biliary damage [106,107]. Moreover, some studies reported GGT as an independent marker for oxidative stress and systemic inflammation [104,107].

Data from our study revealed significant increase in GGT activity in DEN-intoxicated rats compared to normal healthy one. This may be attributed to the rapid turnover of cancer cells that result in releasing of GGT enzyme into the circulation. In accordance with our study, **Salau** et al. [109], found that the liver activity of GGT significantly decreased while an increase in the enzyme activity in serum was detected, suggesting plasma membrane damage caused by injection of DEN. These findings are in agree also with the previous study that showed increase in GGT serum activity, reflecting the oxidative and cellular stress, manifested by depletion in glutathione maintenance inside the cells. **Umarani** et al. [66], indicated that the increase in serum GGT activity in cancer bearing rats may be correlated with the rapid turnover of tumor cells, releasing GGT enzyme into the circulation. This elevation is restored by the effect of Gallic acid administrated to tumor baring rats. Moreover, **Dai** et al. [95]; **Ahmed** et al. [97]; **Hussein and Khalifa** [59]; **Shahat** et al. [98], also reported the same results. This increment in GGT activity indicated the progress of carcinogenesis, as GGT enzyme activity is indicative with the rate of tumor growth [78], also the same authors confirmed this elevation in GGT activity to the up regulation in GGT gene expression level in DEN-intoxicated rats.

On the other hand, the present study showed that GGT inhibiting activity in cisplatin treated rats compared to HCC-bearing rats. This improvement may be resulted from the ability of cisplatin to repair the hepatic damage caused by DEN. Thus, the plasma membrane retains its strength. The improvement in GGT level is also confirmed by the normalization in glutathione level. Also, **Abdel-Hamid** et al. [76] observed that after IP injection with DEN there was marked elevation in GGT enzyme activity, this elevation is reduced post treatment with cisplatin. This may be due to the decrease in oxidative stress caused by DEN. In accordance with the present study **Hassanen** et al. [110], declared that rats injected by DEN followed by CCl_4_ and treated with 1.5 mg/kg cisplatin reflected inhibition in GGT activity compared to untreated rats. On the other hand, **Michael** et al. [111] used cisplatin in the treatment of patients bearing locally advanced and metastatic non-small-cell lung cancer (NSCLC), demonstrated significant improvement in the enzyme level compared to untreated patients.

Using cyclophosphamide in the treatment of DEN/CCl_4_-intoxicated rats also showed inhibition in GGT activity compared to hepatoma bearing group. Our results are run in parallel with **Balasubramaniam** et al. [112]; **Gupta** et al. [113] who attributed the inhibition in GGT activity to cyclophosphamide action on the apoptotic cells.

Our study observed that an improvement in GGT activity post treatment of HCC-bearing rats with 5-FU compared to DEN/CCl_4_-treated rats. This improvement may be a leading cause of anticancer properties of the drug and its ability to adjust the uncontrolled proliferation of cancer cells, thus ameliorate the hazard damage of cells caused by DEN/CCl_4_ induced oxidative stress.

**Mohamad** et al. [114] in their experimental study used 5-FU, oxaloplatin and tamoxifen as a treatment protocol for HCC patients with vitamin E and detected significant inhibition in GGT activity post treatment. In the same regard, **Umarani** et al. [66] also found decline in enzyme activity after treatment with 5-FU compared to cancer induced rats. This may be attributed to a decrease in cell turnover resulting in minimization in the release of the enzyme into the circulation. The current results revealed that supplementation with honey caused significant decrease in GGT activity either used alone or with other chemotherapeutical drugs. This may be revealed to the antioxidant and antiproliferative properties of honey which are able to decrease the hepatocarcinogenic effect of DEN [33]. Moreover, honey retains the cell membrane integrity because of its hepatoprotective effect [115].

In agreement with our study, **Yaman** et al. [51] suggested that the hepatoprotective role of honey against carcinogenic aflatoxin exposure. This was confirmed by not only depletion of GGT activity in honey-treated group compared to HCC-bearing rats, but also it restored the enzyme activity to its normal level. **Tamuno-Emine and Anyia** [116] also observed depletion in GGT activity in honey treated rats compared to cadmium-induced hepatotoxicity in rats. This may be related to its availability of bioflavonoids and the micronutrients as vitamin A, E and C, copper and fructose that protects against cadmium damage. Further, **Abdulrahman** et al. [117] declared that honey supplementation to Egyptian children bearing hepatitis A virus decreased GGT activity and accelerated the recovery as compared to untreated children which reflects the hepatoprotective role of honey.

The present results are in accordance with **Shati and Alamri** [118] who displayed that honey minimized the hepatotoxicity induced by aluminum which is confirmed by measuring many biochemical parameters as GGT. Moreover, **Omnia** et al. [119] demonstrated decline in GGT in rats with induced hyperammonemia treated with propolis compared to untreated rats.

Our study was supported by the histopathological examination of livers in experimental rats. All the biochemical changes post intoxication with DEN/CCl_4_ was proved by the histopathological investigations of liver sections among DEN/CCl_4_-intoxicated rats which showed a proliferation of the hepatocytes with cytoplasmic edema, apparent cellular damage and death. Furthermore, the normal shape and arrangement of hepatocytes are lost, along with vacuoles with different sizes and shapes, necrotic areas with mild cytoplasm, while the nuclei lost their vesicular appearance and became hyper-chromatic. This deterioration may account for the excessive free radicals because of DEN metabolism that caused HCC. In a good agreement with the present findings, **Hussain** et al. [75]**; Zhao** et al. [120]; **Kadasa** et al. [60]; **Chidamabaram** et al. [121]; **Vedarethinam** et al. [122], investigated that DEN-treated rats showed an unformatted architecture, the presence of inflammatory cells along the central vein and enlarged nuclear size in the liver cells.

Treatment of DEN/CCl_4_-intoxicated rats with cisplatin showed less deposition of collagen fibers, binucleation, necrosis of hepatocytes compared to HCC-bearing animals. However, supplementation with honey along with cisplatin exhibited marked improvement in the cell architecture. This is attributed to the anti-proliferation effect of honey against induced HCC. Our result agrees also with **Abdel-Hamid** et al. [76], **Hemieda** et al. [123].

Using cyclophosphamide in treatment of HCC induced rats showed less cytoplasmic vacuolization of hepatocytes and decrease in the collagen deposition compared to DEN/CCl_4_-injected rats. On the other hand, addition of honey to the treatment protocol caused less congestion of central vein and obvious improvement in the cell structure compared to cyclophosphamide treated rats. This may result from the antioxidant effect of honey that reinforces the anticancer effect of the drugs. The study of **Gupta** et al. [118], **Ramakrishnan** et al. [124] also confirmed this improvement in the cell structure after treatment with cyclophosphamide.

The administration of 5-FU to HCC-bearing rats illustrated decrease in Kupffer cells activation, hepatic necrosis and vacuolization of hepatocytes compared to untreated rats. While, treatment with both honey and 5-FU showed improvement in cell structure with deficient collagen deposition. Our findings are in accordance with **Abdel-Hamid** et al. [76], **Cheng** et al. [125].

Although in early stages of HCC, surgery is the main effective and curative treatment option, up to the recent sizeable evidence that honey demonstrates natural immune booster, antioxidant, anti-inflammatory, antimicrobial and most interestingly as cancer vaccine, we can suggest its protective effects against cancer recurrence frequently induced post-surgery by inflammation, oxidative stress, suppressed immune response as risk factors in cancer patients.

## 5 Conclusion

This full scientific and statistical analysis worthy revealed that honey supplementation showed the highest percentages of improvement in AFP, AFU as well as liver function enzymes followed by cisplatin chemotherapeutic drug. In addition, honey administered to carcinogenic rats declared the highest percentages of improvement in CAT, SOD, GPx GST, and oxidative stress biomarker; MDA which correlated well with its antioxidant content. Hence, addition of honey to HCC treatment protocol either alone or in combination with chemotherapeutic drugs, improved the effect of drugs and minimize their side effects. Finally, AFP, AFU and GGT are considered as promising markers for early detection of hepatic damage and treatment evaluation.

## Declaration of Competing Interest

We wish to confirm that there are no known conflicts of interest associated with this publication.
